# Factors Predicting Training Delays and Attrition of Recruits during Basic Military Training

**DOI:** 10.3390/ijerph19127271

**Published:** 2022-06-14

**Authors:** Jamie L. Tait, Jace R. Drain, Sean Bulmer, Paul B. Gastin, Luana C. Main

**Affiliations:** 1 Institute for Physical Activity and Nutrition, School of Exercise and Nutrition Sciences, Deakin University, Geelong, VIC 3220, Australia; j.tait@deakin.edu.au; 2 Defence Science and Technology Group, Fisherman’s Bend, VIC 3207, Australia; jace.drain@gmail.com; 3 Centre for Sport Research, School of Exercise and Nutrition Sciences, Deakin University, Geelong, VIC 3220, Australia; bulmerse@deakin.edu.au; 4 La Trobe Sport and Exercise Medicine Research Centre, School of Allied Health, Human Services and Sport, La Trobe University, Bundoora, VIC 3086, Australia; p.gastin@latrobe.edu.au

**Keywords:** discharge, injuries, stress, physical training, monitoring, soldier, army

## Abstract

Ensuring a balance between training demands and recovery during basic military training (BMT) is necessary for avoiding maladaptive training responses (e.g., illness or injury). These can lead to delays in training completion and to training attrition. Previously identified predictors of injury and attrition during BMT include demographic and performance data, which are typically collected at a single time point. The aim of this study was to determine individual risk factors for injury and training delays from a suite of measures collected across BMT. A total of 46 male and female recruits undertaking the 12-week Australian Army BMT course consented to this study. Injury, illness, attrition, and demographic data were collected across BMT. Objective measures included salivary cortisol and testosterone, step counts, cardiorespiratory fitness, and muscular endurance. Perceptions of well-being, recovery, workload, fatigue, and sleep were assessed with questionnaires. Baseline and mean scores across BMT were evaluated as predictors of injury and attrition using generalized linear regressions, while repeated-measures ANOVA was used for the group comparisons. From the 46 recruits, 36 recruits completed BMT on time; 10 were delayed in completion or discharged. Multiple risk factors for injury during BMT included higher subjective ratings of training load, fatigue, and stress, lower sleep quality, and higher cortisol concentrations. Higher ratings of depression, anxiety, and stress, and more injuries were associated with a higher risk of delayed completion. Higher concentrations of testosterone and higher levels of fitness upon entry to BMT were associated with reduced risk of injury and delayed completion of BMT. Ongoing monitoring with a suite of easily administered measures may have utility in forewarning risk of training maladaptation in recruits and may complement strategies to address previously identified demographic and performance-based risk factors to mitigate injury, training delays, and attrition.

## 1. Introduction

Basic military training (BMT) prepares military recruits for occupational demands by exposing them to physically and cognitively demanding activities and simulations [1]. However, balancing stressor exposure and adequate recovery during this period is necessary to avoid a cascade of maladaptive responses which may lead to injury, illness, and decreased performance [1,2,3,4,5,6], all of which can result in training attrition or discharge. Managing allostatic load is challenging in the BMT context given the fixed training program and group training environment combined with a heterogenous recruit population. Results from the U.S. Army illustrate this challenge, at least in part, by demonstrating that injury risk is highest during BMT and trade-specific training compared with active duty [7]. Furthermore, an examination of the Australian Army BMT course almost 30 years ago showed an attrition (i.e., discharge) rate of 14% [8]. While recent unpublished findings indicate that the discharge rate from the current 12-week BMT course is approximately 10%, an additional 9% of recruits experienced delayed graduation from BMT (i.e., greater than 12 weeks in training, achieved through what is known as ‘backsquadding’). Delays in training have downstream impacts on workforce supply, increase training costs and training inefficiency (i.e., training wastage), and potentially impact career progression [9,10,11].

Understanding and identifying the antecedents of training delays and attrition may allow staff to monitor recruits and detect signs of maladaptation before they manifest as diminished health, performance, musculoskeletal injury, or attrition. Several risk factors have been consistently identified that predict premature attrition and injury during BMT, with these factors often overlapping across both adverse outcomes. These include sustaining a musculoskeletal injury during training [12,13,14], injury history prior to military training [15,16,17,18,19], lower levels of muscular strength and endurance [8,12,13,17,19,20,21], and demographic factors such as older age [19,22], female sex [13,18,22], lower education level [12,15], and smoking status [13,14]. However, these risk factors are typically only measured once, at recruit entry to BMT. 

In sporting contexts, subjective well-being has been used to indicate training status, response to training loads, and recovery [23,24], with negative psychological symptoms (e.g., depressed mood) observed during periods of inadequate recovery and/or intensified training demands. The transition from civilian life to BMT, and the need to meet various training standards to graduate from BMT, can increase psychological distress in recruits [4,25,26], with the potential to contribute to recruit dropout [27,28,29]. Furthermore, negative psychological and behavioural factors such as history of depression, poor mental health and higher stress levels during training have been shown to predict attrition in a few studies [14,15,30]. However, there is a lack of data as to whether subjective ratings of well-being, stress, or even sleep can predict recruit training delays or attrition. These markers may have utility alongside physiological and demographic factors as predictors of attrition, and they may act as surrogate markers of undesirable training loads that may lead to injury, in line with Andersen and Williams’s [31] model of stress and injury. 

Taking a dual approach in measuring a suite of subjective and objective variables in response to BMT over the course of a 12-week program, via serial measurement, may allow a more nuanced analysis, compared with either data that are collected at one time point upon entry to BMT or historical data. Furthermore, although predictors of attrition during BMT have been investigated, these may not align with factors that may also explain delayed progression during BMT [8,12,13,19]. The current study offers a valuable opportunity to differentiate between recruits who completed BMT on time and those who experienced delays in training or were discharged based on their subjective and objective responses. Identifying a suite of measures that may have utility in detecting recruits who may be vulnerable to training delays (inclusive of injury) or attrition is required to: (1) provide targets of assessment for training readiness and (2) underpin decisions on whether countermeasures should be applied to mitigate risks in particular circumstances. Therefore, the main aims of this study were to (1) determine the individual risk factors for training delays or discharge during a 12-week BMT course and (2) compare the responses of recruits who completed a 12-week BMT course on time with the responses of those who did not complete on time. 

## 2. Materials and Methods

### 2.1. Participants

A total of 48 Australian Army recruits (39 men and 9 women) commencing the 12-week BMT program at the Army Recruit Training Centre (Blamey Barracks, Kapooka) in July 2018 were recruited for this study. The recruits were from two platoons within the same training company and commenced BMT on the same day. Of these 48, *n* = 38 completed BMT on time; two recruits were removed from analysis due to insufficient data (<80% of data), which was the threshold for inclusion. Thus, the final sample size for analysis was *n* = 46, comprising *n* = 36 who completed BMT on time and *n* = 10 who did not complete on time due to backsquadding (*n* = 7) or due to discharge (*n* = 3). Recruits were briefed on the investigation at the commencement of BMT, prior to providing voluntary, written informed consent. This study was approved by the Department of Defence and Veteran’s Affairs Human Research Ethics Committee (021-17). An overview of activities completed during BMT has been previously described [32,33], while sleep duration for these recruits has also been reported [33]. Activities included training related to military skills and equipment, marksmanship, and combat manoeuvres, and culminated in a 10-day field training phase which involved sleep restriction, physical and cognitive demands, and outdoor sleeping and night activities (e.g., picket). 

### 2.2. Anthropometry and Body Composition

Height was measured to the nearest 0.1 cm, and body weight was measured to the nearest 0.01 kg using standard techniques (stadiometer and metric scale, respectively). Body mass index (BMI) was calculated [weight (kg)/height (m^2^)].

### 2.3. Injury, Illness and Training Outcomes

Injury, illness, and training outcome data (on-time BMT completion, delayed BMT completion, discharge) were obtained from local records at the Army Recruit Training Centre. Injuries were classified according to body region/area affected [34]. Lower limbs included: the hip, thigh, knee, ankle, and foot, while the trunk was classified as the chest and back. Injury type was classified as strain/sprain, which referred to ligamentous injury or an injury to the musculotendinous complex; stress fracture was indicated as a bone stress injury. Causal action data were also collected, although all injuries were regarded as strain in type, except for gradual onset (*n* = 1) and acute trauma from a falling object (*n* = 1). The activity being undertaken at the time of injury was also recorded. 

### 2.4. Salivary Cortisol and Testosterone

Salivary testosterone and cortisol were collected weekly via salivette. To control for the diurnal rhythm, participants provided saliva samples upon waking, 30 min post-waking, and immediately before bed. Participants were instructed to refrain from eating or drinking before sampling and to avoid alcohol for at least 12 h prior to sample collection to reduce the contamination of samples. The samples were initially stored at Kapooka Health Centre in a −20 °C freezer and then transported by car to Deakin University, where they were stored (in a −80 °C freezer) until analysis. For analysis, all salivettes were centrifuged for 10 min at 1000 g before saliva was aliquoted and frozen at −20 °C. Biomarkers were analysed in duplicate. Saliva cortisol and testosterone were measured using ELISA kits (IBL International, Hamburg, Germany) per manufacturers’ recommendations. All analyses recorded an intra-assay % coefficient of variation (%CV) of <8% and an inter-assay %CV of <19%.

### 2.5. Well-Being: Depression Anxiety and Stress Scale (DASS-21)

Symptoms of depression, anxiety, and stress were assessed at weeks 1, 4, 8, and 12, using the Depression Anxiety and Stress Scale (DASS)-21 [35], a 21-item self-report measure, with 7 items on 4-point Likert scales for each factor of depression, anxiety, and stress; higher scores indicate greater anxiety, depression, or stress [36].

### 2.6. Short Recovery Stress Scale 

The Short Recovery Stress Scale assesses both the recovery and stress state of an individual at the time of surveying [37]. Recovery-related questions assess physical performance capability, mental performance capability, emotional balance, and overall recovery. Stress-related questions assess muscular stress, lack of activation, negative emotional state, and overall stress. Participants answered eight questions once a week upon waking on Sunday mornings via pen and paper, indicating the extent to which each statement applied to them at the time. Response options range from 0 (does not apply at all) to 6 (fully applies). Ratings from individual items of each subscale were summed to form composite scores for recovery and stress, with higher scores indicating greater recovery and stress respectively. 

### 2.7. Subjective Load and Fatigue 

The following information was recorded via entry into a recording diary each evening before bed, along with potential difficulties with data collection procedures that day. Subjective load was measured via the NASA Task Load Index (NASA-TLX) [38], consisting of one question for each of six subscales measuring levels of mental, physical, and temporal demands, performance, effort and frustration along a continuum, with an integer value of 0–100 in 5-point increments. The NASA-TLX is sensitive for the assessment of cognitive demands of a given workload [38,39]. Participants’ subjective ratings of fatigue (pre-sleep and post-sleep) were assessed via the Samn-Perelli Fatigue Scale, scored on a seven-point Likert scale [40]. Developed and validated in occupational settings such as aviation operations, it is reliable and sensitive to the effects of sleep loss at different times of the day. 

### 2.8. Physical Activity Load

The physical demands of BMT have been previously reported, in regards to intensity and steps [32]. External load was captured via daily physical activity counts recorded with an ActiGraph GT9X (Acti-Graph, Pensacola, FL, USA) set at 30 Hz using 60-s epochs, worn on the non-dominant wrist. The participants wore a device for the entire duration of the study except for any periods of water immersion (i.e., showering or swimming), and to charge the units. These devices were collected by research staff Monday night prior to bedtime; the data were downloaded, and the devices were charged prior to their return to participants upon waking Tuesday morning. Non-wear time was determined as more than three hours of consecutive 0 total acceleration per day, except Monday and Tuesday, where eight hours was used to account for downloading and charging time [41]. The 3-h non-wear threshold is more stringent than the standard minimum 10-hour/day total wear time often used in free living adults. Week one accelerometry data were not utilized in the statistical analysis, as less than four days of data were available after consenting.

### 2.9. Physical Fitness

Physical fitness was assessed via the maximum number of push-ups in 2 min completed according to an audio cadence track (maximum 100), and 20-m multi-stage shuttle test (MSST) performed during weeks 2 and 8 of BMT. MSST performance was used to estimate $\dot{V}$O_2max_ according to Ramsbottom et al. (1988) [42]. 

### 2.10. Statistical Analyses

All statistical analyses were conducted using STATA statistical software release 15.0 (STATA, College Station, TX, USA). All data and their residuals were checked for normality. Participants who completed BMT on time and remained in the study for the 12-week period were classified as ‘on-pathway’ (ON-P), and those who did not complete BMT on time or discharged were regarded as ‘off-pathway’ (OFF-P). For the preliminary analyses, we attempted to gain insight into any differences in the trajectories of objective and subjective responses that may represent risk factors for injury and attrition, between ON-P and OFF-P. Repeated-measures ANOVA were conducted, with group as a fixed effect, time as the repeating unit, and participants included as random effects. The mean weekly concentrations and scores were used in analyses of objective and subjective measures. Post-hoc comparisons were made with Bonferroni corrections for multiple comparisons. As the majority of participants within OFF-P did not provide data to our study beyond week 6, comparisons between groups were analysed up to and including this timepoint.

For the main analyses, to assess predictors of (a) musculoskeletal injury and (b) deviating off pathway (discharge and delayed completion), baseline scores and mean scores across training were used. Push-ups and predicted $\dot{V}$O_2max_ were only collected at baseline. Relative risk (incident risk ratio) for off-pathway and injury was estimated via generalized linear regression, incorporating a log link and Poisson distribution and estimating a robust error variance. Due to their role in potentially explaining variance in risk for off-pathway and injury, sex, mean step count across training, and predicted baseline $\dot{V}$O_2max_ were included as covariates in determining the risk of injury and off-pathway deviation for the selected variables. To identify independent risk factors for injury and off-pathway deviation, individual risk factors significantly predicting injury and off-pathway were then used as covariates using a backward elimination procedure that sequentially removed variables that did not contribute significantly (*p* > 0.05) to explaining the risk of attrition or injury. Factors were separated as to whether they explained either higher risk (RR > 1) or lower risk (RR < 1) for injury and off-pathway. Separate models were subsequently run to assess the factors that independently explained either higher risk or lower risk. As a sub-analysis for attrition, the group OFF-P were further categorized into those who were delayed in their BMT completion due to backsquadding or other factors [denoted as delayed completion (DELCOM)], and those who did not complete BMT because they left the Army (denoted as discharged, DIS). For this, we compared risk ratios for ON-P vs. DELCOM and DIS. All data are presented as means ± SEM or 95% CI unless stated.

## 3. Results

### 3.1. Participants

At baseline (week 1), there were differences in height, push-ups, and steps between ON-P and OFF-P (Table 1). The nature and number of injuries, the reasons for non-timely completion, and the time point of injury and attrition are presented in Table 2 and Table 3. For the six participants who sustained injuries in OFF-P, the consequences were: delayed on march-out (*n* = 5) and discharged (*n* = 1). 

### 3.2. Comparison of Responses between On-Pathway and Off-Pathway

#### 3.2.1. Subjective Measures

**Figure 1:**
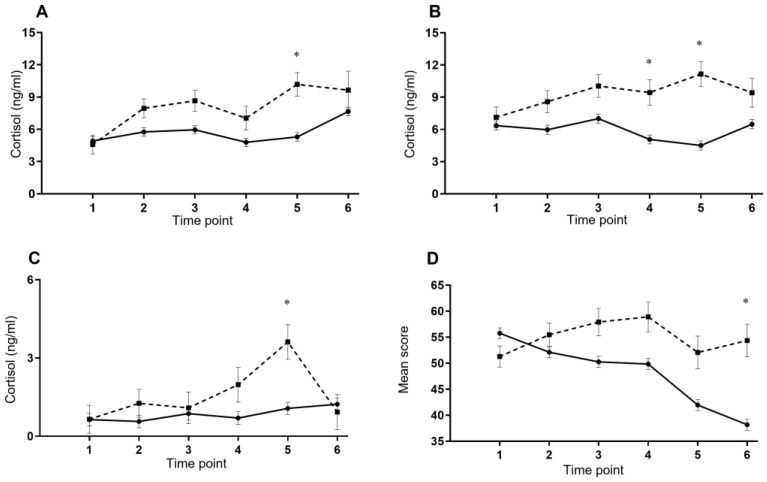
Mean (±SEM) changes in (**A**) waking cortisol concentration, (**B**) 30 min post-waking cortisol concentration, (**C**) bedtime cortisol concentration, (**D**) NASA-TLX six-subscale average during the first 6 weeks of BMT between those who completed BMT on time (ON-P; solid line; —) and recruits who did not (OFF-P; dashed line; - - -) * *p* < 0.05.

There were no between-group differences for the change over time (between week 1 and week 6) for depression, anxiety, and stress subscales of the DASS; the recovery or stress composites; or any fatigue measures. There was a group by time interaction for perceived cognitive load (NASA-TLX average subscale score) (*p* < 0.001; Figure 1), with a significant difference between groups at week 5 (*p* < 0.001). 

#### 3.2.2. Objective Measures

There were no between-group differences for the change over time for testosterone levels, predicted cardiorespiratory fitness, upper body muscular endurance, or step counts. For waking cortisol levels, there was a significant difference between groups for the change over time (*p* = 0.034); compared with ON-P, OFF-P had significantly higher concentrations at week 5 (*p* = 0.03; Figure 1). For 30 min post-waking cortisol levels, there was a significant difference between groups for the change over time (*p* = 0.019), OFF-P had significantly higher concentrations at week 4 (*p* = 0.043) and week 5 (*p* < 0.001; Figure 1) compared with ON-P. For bedtime cortisol levels, there was a significant difference between groups for the change over time (*p* = 0.038): OFF-P had significantly higher concentrations at week 5 (*p* < 0.001; Figure 1).

### 3.3. Predictors of Injury 

#### 3.3.1. Baseline Values as Predictors of Injury Risk

For baseline values, a higher number of push-ups [RR = 0.95 (0.91–0.99), *p* = 0.022] predicted $\dot{V}$O_2__max_ [RR = 0.83 (0.76–0.91), *p* < 0.001] and SRSS Recovery composite [RR = 0.82 (0.72–0.93), *p* = 0.002] were associated with lower risk of injury (Table 4). Independent factors for lower injury risk were higher baseline predicted $\dot{V}$O_2__max_ [RR = 0.81 (0.73–0.90), *p* < 0.001] and SRSS Recovery composite scores [RR = 0.81 (0.68–0.96), *p* = 0.013].

#### 3.3.2. Mean Scores across Training as Predictors of Injury 

Recruits categorized as off-pathway were at 3.26 (1.40, 7.60; *p* = 0.006) times greater risk of sustaining an injury during BMT compared with those who completed BMT on time (Table 4), even after adjusting for sex and step counts 2.84 (1.05, 7.71; *p* = 0.040). Female recruits were at 3.71 (1.63, 8.46; *p* = 0.002) times greater risk of sustaining an injury compared with males, although this was no longer significant after adjusting for predicted $\dot{V}$O_2__max_ (*p* = 0.176). Females sustained more injuries than males; 1.1 injuries per female (10 in total) vs. 0.21 injuries per male (7 in total, *p* = 0.006). Individual risk factors for injury incidence were higher mean scores across training in pre- (*p* = 0.030) and post-sleep fatigue (*p* = 0.002), poorer sleep quality (*p* = 0.016), higher scores on the SRSS Stress composite (*p* = 0.046), NASA-TLX total (*p* = 0.046), and higher concentrations of bedtime cortisol (*p* < 0.001). Higher SRSS Recovery scores (*p* = 0.001) and higher concentrations of testosterone at waking (*p* < 0.001), 30 min post-waking (*p* < 0.001), and at bedtime (*p* = 0.001) were associated with lower risk of injury.

#### 3.3.3. Independent Predictors of Injury 

The results of the multivariate general linear model regression analysis indicated that independent risk factors for greater injury risk were delayed BMT completion [RR = 2.82 (1.16, 6.82); *p* < 0.001], higher pre-sleep fatigue [RR = 1.78 (1.24, 2.55); *p* = 0.002], higher mean bedtime cortisol concentration [RR = 1.92 (1.37, 2.71); *p* < 0.001], higher mean SRSS Stress composite [RR = 1.72 (1.52, 1.96); *p* < 0.001], and higher mean NASA-TLX total [RR = 0.62 (0.52, 0.73); *p* < 0.001]. 

Independent risk factors for lower injury risk were higher mean SRSS Recovery score [RR = 0.84 (0.73, 0.97), *p* = 0.020], higher mean testosterone levels 30 min post-waking [RR = 0.97 (0.94–0.99), *p* = 0.017] and higher predicted $\dot{V}$O_2__max_ [RR = 0.81 (0.67–0.98), *p* = 0.033]. 

### 3.4. Predictors of Deviating Off-Pathway during BMT

#### 3.4.1. Baseline Values as Predictors of Off-Pathway (Delayed March-Out or Attrition) 

For the baseline scores, higher numbers of push-ups were associated with lower risk of deviating off-pathway [RR = 0.95 (0.91–0.99), *p* = 0.012]. 

#### 3.4.2. Mean Scores across Training as Predictors of Off-Pathway (Delayed March-Out or Attrition) 

Recruits who sustained an injury were at 4.03 (1.38, 12.2; *p* = 0.013) times greater risk of deviating off-pathway (OFF-P; Table 4), even after adjusting for sex and step counts 2.84 (1.05, 7.71; *p* = 0.040). Individual risk factors for off-pathway deviation were mean scores for the DASS-21 subscales of depression (*p* = 0.025) and stress (*p* = 0.003), higher mean SRSS Stress composite scores (*p* = 0.024), higher NASA-TLX total scores (*p* = 0.023), and higher concentrations of cortisol at 30 min post-waking (*p* = 0.010). Mean higher waking testosterone concentration (*p* < 0.001), higher SRSS recovery composite scores (*p* = 0.020) and higher step counts were associated with lower risk of being categorized as off-pathway (*p* < 0.001).

#### 3.4.3. Independent Predictors of Off-Pathway (Delayed March-Out or Attrition) 

The results of the multivariate general linear model regression analysis indicated that independent risk factors for off-pathway deviation were injury [RR = 12.3 (4.79, 31.3), *p* < 0.001], higher mean NASA-TLX total [RR = 1.09 (1.03, 1.15); *p* = 0.001], higher mean 30 min post-waking cortisol concentration [RR = 1.36 (1.17, 1.59); *p* < 0.001], and higher scores for DASS stress (RR = 1.21 (1.07, 1.36), *p* = 0.002). Conversely, higher mean testosterone waking levels [RR = 0.98 (0.96–0.99), *p* = 0.028] were associated with decreased risk of off-pathway. 

#### 3.4.4. Risk of Delayed Completion and Discharge for BMT: Off-Pathway Sub Analysis

The baseline characteristics (week 1) for the different subcategories, on-Pathway, Delayed completion, and Discharged, are presented in Supplementary Table S1. Compared with ON-P, individual risk factors for the subgroup who were delayed in their completion of training (DELCOM) were: sustaining an injury (*p* = 0.010), higher mean SRSS Stress composite scores (*p* = 0.041), higher NASA-TLX total scores (*p* = 0.023), and higher concentrations of cortisol at 30 min post-waking (*p* = 0.047; Table 5). Individual risk factors for being discharged were higher mean scores for the DASS-21 subscales of depression (*p* = 0.018), stress (*p* = 0.039), and anxiety (*p* = 0.028). 

## 4. Discussion

This study identified risk factors for injury, delayed completion and attrition during the Australian Army BMT program. Higher subjective ratings of training load, fatigue, and stress and lower subjective sleep quality were associated with a higher risk of injury, while higher levels of depression, anxiety, and stress were associated with a greater risk of delayed march-out or discharge. Increased salivary cortisol was associated with a higher risk of injury, while higher testosterone was associated with a decreased risk of injury and training ‘wastage’ (i.e., delayed BMT completion, discharge). Higher levels of fitness upon entry to BMT may also protect against injury and delayed completion of BMT, while the incidence of injuries predisposed recruits to a higher risk of delayed completion and/or discharge. The serial measurement of these subjective and objective markers could potentially be used in larger monitoring systems to indicate whether recruits are coping with training demands, and the data could signal those at risk of negative training outcomes (e.g., injury, delayed BMT completion, discharge). 

### 4.1. Risk Factors of Injury 

Our study was novel in that we used data collected across BMT to identify a suite of subjective and objective measures associated with risk of recruit injury and attrition which may be used to monitor recruit training states across BMT. We observed that females sustained more injuries and had a higher injury rate than males (1.1 vs. 0.21 injuries/recruit); however, after adjusting for cardiorespiratory fitness, there was no difference in injury risk between sexes. This is consistent with recent investigations in both recruits [43] and active-duty personnel [44]. Several risk factors have been associated with increased risk of injury during BMT, including pre-BMT injury history [17,18], female sex [18,22], and physical fitness [8,12,17,21,44]. However, these measures are typically measured once, upon recruit entry to BMT, and not adjusted for factors that may also influence injury risk. We observed that subjective measures such as greater pre- and post-sleep fatigue, decreased sleep quality, greater perceptions of physical training stress, and cognitive load were associated with a higher risk of injury. In particular, the risk of injury was twice as high in those with higher ratings of fatigue and poorer sleep quality across BMT. The mechanisms underpinning these findings are unclear, although poor sleep patterns (e.g., disturbances, sleep loss, poor sleep quality) are associated with an increased risk of occupational injury [45,46], with affected workers having a 1.62 times higher risk of being injured than workers without sleep problems [45]. In military settings specifically, fatigue can increase accidents and errors in judgement and interpretations [47]. This may further link with theoretical models of stress and injury [31], which speculate that individuals with higher levels of stress are at a greater risk of injury and accidents, due to increased muscle tension, peripheral narrowing, and increased distractibility, confirmed in other research [48]. 

High workloads, insufficient recovery opportunities, and increased stress during military training have been linked to a number of maladaptive outcomes, such as musculoskeletal injury, illness, and training attrition [49]. We observed that training load, objectively measured as step count, was not a significant predictor of injury or deviating off-pathway. This may result from recruits undertaking a similar training program, with minimal variation in load. Conversely, subjective measures of load and perceptions of recovery and fatigue were linked to a greater risk of injury, delayed completion, and attrition. These data suggest that the coping strategies employed by recruits during BMT may moderate their responses to the training load and partially contribute to training success or discontinuation. We also showed for the first time that higher concentrations of salivary cortisol were associated with greater risk of negative training outcomes (injury, delayed completion, and discharge). This infers that the presence or upregulation of catabolic processes may be a predisposing factor for adverse training outcomes. The risk of injury was twice as high in those with higher concentrations of bedtime cortisol. Cortisol has been shown to be a bio-behavioural indicator of training strain in military contexts [50,51] and is regarded as a classic marker of psychological stress [52], which may link to injury [31]. In addition, we observed that higher concentrations of salivary testosterone and greater perceptions of recovery were associated with a decreased risk of injury and being off-pathway, which suggests that an anabolic state, sufficient recovery opportunities, or at least the perception of adequate recovery may mitigate negative training outcomes. The periodization of recovery periods found within the current BMT course may explain the associations between higher perceptions of recovery, and higher concentrations of testosterone, with lower injury risk and attrition. In particular, increases in or maintained levels of testosterone have benefits for maintaining bone and muscle mass and for positive adaptations to training stimuli [53]. A lower number of injuries in recruits would reduce the risk of delayed march-out or discharge. Strategies that can mitigate excessive strain and perceptions of load may be useful in reducing the risk of injury, particularly as stressors throughout BMT can cumulatively exceed coping resources. 

Lower pre-BMT levels of muscular strength and endurance [17,21] and cardiorespiratory fitness [8,17,20], have been associated with a greater risk of injury in recruits. In support of this previous research, the current investigation similarly found that lower baseline cardiorespiratory fitness and muscular fitness were associated with a higher risk of injury during BMT. In addition, 53% of all injuries occurred within the first four weeks of BMT, with more than half (59%) of all reported injuries across BMT occurring in the lower limbs. Collectively, these results suggest that the physical activity exposure, inclusive of physical training, during the initial weeks of BMT exposes recruits with lower physical fitness to increased injury risk. Future research could investigate the efficacy of pre-conditioning strategies to improve fitness prior to commencing BMT in order to reduce the strain experienced during BMT and improve training outcomes. 

### 4.2. Risk Factors of Delayed March-Out and/or Discharge 

Some attrition from BMT is inevitable as not all recruits will cope well with the physical and/or psychological demands. Our findings suggest that the monitoring of well-being and hormone levels may be beneficial for flagging individuals at increased risk of injury and attrition. Previous research has indicated that BMT is a psychological stressor [4,25] that produce behavioural symptoms of overtraining in recruits (e.g., confusion, depression, fatigue) [4]. However, some authors have reported improvements in scores for depression, anxiety and stress subscales across BMT [26,54], ascribed to successful integration into the training program and adequate recovery opportunities. We observed that higher ratings of depression, anxiety. and stress were associated with a ~10% greater risk of a delayed march-out/discharge from BMT. Poorer well-being was also specifically associated with discharge from BMT, albeit in a small subsample (*n* = 3). However, as a caveat, the majority of those who had a delayed march-out or were discharged had stopped providing data by week 6, which limits the generalizability of our findings. Previous research has suggested that levels of depression, anxiety, and stress are elevated at the start of BMT due to the unfamiliar and challenging conditions of military training and low confidence, which gradually subsides over the course of training [26,55,56]. In the current study, there was a significant divergence in cognitive load across the first six weeks, when comparing those who were on-pathway with those who were off-pathway. Moreover, those not completing BMT on time exhibited perceptions of load in the initial weeks that increased, before a subsequent decrease in rating, compared with those completing on time who exhibited declines after the first week. Perceptions of greater physical training stress and cognitive load were additionally associated with a higher risk of not completing BMT on time. Therefore, minimizing depressed moods and enhancing perceived well-being prior to and during BMT (e.g., through stress resistance strategies) may improve BMT outcomes but also better prepare recruits for the stressors encountered during a military career.

Given that a higher number of injuries was also associated with a greater risk of delayed march-out or discharge, it is unclear if higher ratings for depression and stress are corollaries of injury incidence and can lead to training attrition or whether stress and injury interact to compound the risk of attrition. Those with delayed march-out or attrition also exhibited higher concentrations of morning cortisol across weeks 1–6, compared with those who completed the study, which may suggest an elevated stress response. Increases in waking salivary cortisol concentration have been reported in depressed individuals [57], in response to psychological stress and anxiety [58], and in conjunction with repeated episodes of psychological stress and/or burn-out [59]. This suggests that hypothalamic-pituitary axis dysregulation may be related to poorer mood, lower ratings of well-being and higher levels of perceived stress, all of which can compromise performance and training progression. The measurement of cortisol in saliva therefore provides a reliable tool for investigations of HPA activity and could be measured in a serial manner over the course of BMT, to flag those who require well-being intervention, and ultimately mitigate the risk of delayed march-out and training attrition. 

Levels of muscular fitness may also influence the timely completion of BMT through direct links with injury risk and indirect associations with well-being. We observed that a higher level of muscular fitness reduced the risk of delayed completion of BMT. Previous research has proposed that well-being or perceptions of stress during BMT may be shaped by factors such as fitness levels, resiliency, and hardiness [60,61,62], and these factors may further interact to influence attrition risk. Physical fitness may have protective effects in buffering the adverse effects of stress [63,64], while physical training may confer benefits to well-being through increased self-efficacy and self-esteem [65], which may in turn reduce the risk of attrition. Given the proposed relationships between greater physical fitness and increased resiliency in soldiers [66], the associations between well-being, fitness levels, and attrition require further evaluation. 

Collectively, close attention is required to monitor recruits across the first four to six weeks, when the majority of injuries and off-pathway deviations occurred for this cohort, to identify those who are not responding well to the training program. Early identification of these individuals at risk may allow for appropriate risk mitigation strategies to avoid injury and attrition. Risk mitigation strategies may include counselling, targeted changes to the BMT program for individuals or strategic backsquadding to reduce the risk of serious injury or discharge. Additional strategies may include early education or intervention with mental skills training to improve recruit resilience and introduce coping strategies, which may assist with recruit expectation management and self-perceptions of fatigue, recovery, and coping. 

Based on estimates from 1996, the current cost associated with enlistment and training an Australian Army recruit is approximately $47,000 [67], which is consistent with U.S. data [7]. This estimate has been utilized to understand the direct economic cost of training wastage during the Australian Army BMT. Rates of 10% attrition and 9% delayed completion results in an estimated cost of $5.16 M per 1000 recruits (Supplementary Table S2). It is acknowledged that this projected cost may not be entirely accurate, but the authors suggest that this nonetheless helps to illustrate the cost of training wastage, especially considering that musculoskeletal injury is a major cause of both discharge and delayed completion [12,13,14], and chronic overuse injuries comprise the majority of injuries. Therefore, strategies such as pre-enlistment pre-conditioning strategies and well-being monitoring during BMT have potential utility in decreasing preventable training wastage.

### 4.3. Strengths/Limitations

There are a number of strengths associated with this study. To the best of our knowledge, this is the first study to conduct serial measurement of objective and subjective markers during BMT and to evaluate their associations with injury and non-timely completion of BMT. There are also limitations which must be considered when interpreting the results. Due to the study aims, factors known to influence hormonal levels were not included (e.g., pre-enlistment activity history, nutritional status), but it is acknowledged they may have influenced findings [68]. Second, as we used an average of ratings and levels across BMT, it is not possible to determine the periods or activities within BMT that predispose recruits to a greater risk of attrition or injury. Third, a focus of the study was determining the demographic, fitness, and subjective factors that may be useful in determining the risk of injury and attrition, with the inclusion of salivary hormones an exploratory opportunity. However, the majority of recruits in the DELCOM and DIS groups stopped providing study data by week 6, and a small number from those groups did not provide any data. For example, step counts and hormone samples may only have been obtained from in weeks 1–4. For this reason, the generalizability of the attrition data, compared with those remaining in the study with all 12 weeks of data, should be confirmed in future studies.

## 5. Conclusions

In conclusion, a low-burden suite of objective and subjective measures appears to be useful in monitoring training demands of recruits that if implemented could minimize risk of injury and attrition. Salivary cortisol was associated with a higher risk of injury, while higher amounts of testosterone may be useful indicators of lower risk of injury and the potential to complete BMT on time. Higher levels of fitness upon entry to BMT may also protect against injury and attrition, while more injuries predisposed recruits to a higher risk of not completing BMT in a timely manner and/or discharge. Higher subjective ratings of training load, fatigue, stress, and lower sleep quality were associated with a higher risk of injury, while higher levels of depression, anxiety, and stress were associated with a greater risk of delayed completion and/or discharge. Based on our overarching findings, future work should explore avenues and countermeasures that develop and preserve cardiorespiratory fitness, strength, and well-being and accelerate perceptions of recovery. Serial measurement of these markers could potentially be used in larger monitoring systems to indicate whether recruits are coping with the training stress, and signal those at risk of injury and attrition.

## 6. Practical Applications

Perceptual factors should be considered in conjunction with objective measures in personnel management decisions. Easily administered subjective measures and fitness measures (e.g., shuttle run, push-ups) at baseline, and monitored throughout BMT, appear to be effective markers for a recruit’s susceptibility to injury and attrition, which by extension may infer training readiness. 

Understanding and identifying the factors that precipitate injury and attrition may allow staff to better monitor recruits and detect signs of maladaptation, providing the opportunity to intervene before adverse outcomes occur. This may include a slight increase in the sleep opportunity for a given night or two, or a subtle reduction in some individuals’ training loads in some way (i.e., sets/reps in physical training). 

Levels of cardiorespiratory fitness and upper body muscular endurance at the commencement of BMT should be enhanced to mitigate injury risk, which suggests that pre-conditioning programs may play a role. 

## Figures and Tables

**Table 1:** Recruit characteristics at week 1 of BMT.

Characteristics	On-Pathway	Off-Pathway
N	36 (78%)	10 (22%)
Discharged		3 (7%)
Delayed march-out		7 (15%)
Men, *n* (%)	31 (86%)	6 (60%)
Women, *n* (%)	5 (14%)	4 (40%)
Age, year	24.1 ± 6.8	26.4 ± 8.4
Height, cm	178.0 ± 9.7 *	169.4 ± 9.8
Weight, kg	76.0 ± 14.6	72.7 ± 15.8
BMI (kg/m^2^)	23.9 ± 3.3	25.0 ± 3.1
Injuries, *n*	11	6
Recruits with injuries, *n*	6 (16.7%)	6 (60%)
Objective measures		
Predicted $\dot{V}$O_2max_, mL·kg^−1^·min^−1^	43.2 ± 4.4	40.5 ± 3.6
Push-ups completed, *n*	32.8 ± 10.9 *	23.9 ± 13.0
Cortisol (ng/mL)		
Waking	4.9 ± 1.9	4.6 ± 2.8
30 min post-waking	6.4 ± 2.4	7.1 ± 3.0
Bedtime	0.6 ± 0.4	0.7 ± 0.4
Testosterone (pg/mL)		
Waking	180.4 ± 51.5	156.7 ± 78.8
30 min post-waking	158.6 ± 45.9	167.7 ± 76.7
Bedtime	109.6 ± 41.8	104.1 ± 35.9
Steps per week, n	108,608 ± 16,074 *	82,077 ± 26,049
Subjective measures		
Short Stress Recovery Scale		
Stress Composite	7.7 ± 4.0	10.3 ± 4.9
Recovery Composite	13.8 ± 3.8	11.0 ± 3.0
NASA-TLX Average	55.4 ± 9.3	51.3 ± 12.6
Fatigue		
Pre-sleep fatigue	4.0 ± 1.0	3.8 ± 1.0
Post-sleep fatigue	3.9 ± 1.0	3.8 ± 0.9
DASS-21		
Depression	7.2, 4.0 ± 10	9.5, 5.0 ± 15
Anxiety	8.9, 8.0 ± 12	8.8, 6.0 ± 12
Stress	12.2, 10.0 ± 16	16.0, 14.0 ± 9

Note: Values are mean ± SD except for DASS-21: mean, median ± interquartile range. DASS: Depression Anxiety and Stress Scale; off-pathway: did not complete basic military training (BMT) on time or were discharged, on-pathway: completed BMT on time; PSQI: Pittsburgh Sleep Quality Index; NASA-TLX: NASA Task Load Index; * *p* < 0.05 compared to off-pathway.

**Table 2:** The numbers and percentages of musculoskeletal and health complaints reported by the participants during BMT.

Body Region	Nature of Injury/Complaint	Complaints, *n* (%)
Lower limbs	Strain/sprain	9 (52.9%)
	Stress fracture	1 (5.9%)
Trunk (incl.back)	Strain/sprain	4 (23.5%)
Other	Undefined illness/injury	3 (17.6%)
	Total	17 (100%)
Activity at time of injury		
Physical Training	Strength training	5 (29.4%)
	Swimming	1 (5.9%)
	Running	2 (11.8%)
General Training	Pack marching	1 (5.9%)
	Training activities	2 (11.8%)
	Field training	1 (5.9%)
	Unloading/picking up equipment	2 (11.8%)
Other	Undefined activity	3 (17.6%)
	Total	17 (100%)

**Table 3:** The time points of musculoskeletal injuries and the off-pathway incidence during BMT.

BMT Week	Injuries, *n* (%)	Injuries, Cumulative Total (%)	Off-Pathway, *n* (%)	Off-Pathway, Cumulative Total (%)
**Week 1**	1 (5.9%)	1 (5.9%)	0 (0)	0 (0%)
**Week 2**	3 (17.6%)	4 (23.5%)	2 (20%)	2 (20%)
**Week 3**	1 (5.9%)	5 (29.4%)	2 (20%)	4 (40%)
**Week 4**	4 (23.5%)	9 (52.9%)	1 (10%)	5 (50%)
**Week 5**	0 (0%)	9 (52.9%)	0 (0%)	5 (50%)
**Week 6**	0 (0%)	9 (52.9%)	0 (0%)	5 (50%)
**Week 7**	0 (0%)	9 (52.9%)	2 (20%)	7 (70%)
**Week 8**	1 (5.9%)	10 (58.8%)	1 (10%)	8 (80%)
**Week 9**	0 (0%)	10 (58.8%)	1 (10%)	9 (90%)
**Week 10**	1 (5.9%)	11 (64.7%)	1 (10%)	10 (100%)
**Week 11**	4 (23.5%)	15 (88.2%)	0 (0%)	10 (100%)
**Week 12**	0 (0%)	15 (88.2%)	0 (0%)	10 (100%)
**Undefined**	2 (11.8%)	17 (100%)	0 (0%)	0 (0%)
**Total**	17 (100%)		10 (100%)	

**Table 4:** Factors explaining the risk of recruit injury and off-pathway deviation during BMT.

	Injury Risk		Risk of Off-Pathway	
Factors	Risk Ratio (95% CI)	*p* Value	Risk Ratio (95% CI)	*p* Value
Sex, Females	3.71 (1.63, 8.46)	*p* = 0.002	2.89 (1.01, 8.24)	*p* = 0.047
Non-completion	3.26 (1.40, 7.60)	*p* = 0.006	-	
Injury	-		4.03 (1.38, 12.2)	*p* = 0.013
Pre-sleep fatigue	1.63 (1.05, 2.55)	*p* = 0.030	0.95 (0.53, 1.71)	ns
Post-sleep fatigue	2.02 (1.30, 3.14)	*p* = 0.002	1.42 (0.79, 2.52)	ns
Sleep quality	2.15 (1.15, 3.99)	*p* = 0.016	1.26 (0.61, 2.58)	ns
SRSS: Stress	1.04 (1.00, 1.09)	*p* = 0.046	1.06 (1.01, 1.11)	*p* = 0.024
SRSS: Recovery	0.80 (0.69, 0.91)	*p* = 0.001	0.85 (0.74, 0.97)	*p* = 0.020
DASS: Depression	0.98 (0.91, 1.04)	ns	1.07 (1.03, 1.12)	*p* = 0.025
DASS: Anxiety	0.99 (0.91, 1.09)	ns	1.06 (0.97, 1.16)	ns
DASS: Stress	1.04 (0.97, 1.11)	ns	1.11 (1.04, 1.19)	*p* = 0.003
NASA-TLX: Subscale Average	1.04 (1.00, 1.09)	*p* = 0.046	1.06 (1.01, 1.11)	*p* = 0.023
Cortisol: waking	1.10 (0.94, 1.28)	ns	1.10 (0.89, 1.36)	ns
Cortisol: +30 min	1.11 (0.96, 1.29)	ns	1.27 (1.09, 1.46)	*p* = 0.002
Cortisol: bedtime	2.03 (1.49, 2.76)	*p* < 0.001	1.77 (0.93, 3.36)	ns
Testosterone: waking	0.99 (0.97, 0.99)	*p* < 0.001	0.99 (0.98, 0.99)	*p* = 0.010
Testosterone: +30 min	0.98 (0.97, 0.99)	*p* < 0.001	0.99 (0.98, 1.00)	ns
Testosterone: bedtime	0.98 (0.96, 0.99)	*p* = 0.001	0.99 (0.97, 1.01)	ns
Step count	0.99 (1.00, 1.00)	ns	0.99 (0.99, 0.99)	*p* = 0.002

Note: DASS: Depression Anxiety and Stress Scale; NASA-TLX: NASA Task Load Index; SRSS: Short Recovery Stress Scale. ns = not significant.

**Table 5:** Factors explaining the risk of delayed completion of BMT.

	Delayed Completion, *n* = 7		Discharge, *n* = 3	
Factors	Risk Ratio	*p*-Value	Risk Ratio	*p*-Value
Sex, Females	2.64	ns	13.2	ns
Injury	11.1	*p* = 0.010	2.21	ns
Pre-sleep fatigue	0.83	ns	1.33	ns
Post-sleep fatigue	1.32	ns	2.44	ns
Sleep quality	0.98	ns	2.8	ns
SRSS: Stress	1.10	*p* = 0.041	1.05	ns
SRSS: Recovery	0.80	ns	0.83	ns
DASS: Depression	1.02	ns	1.23	*p* = 0.018
DASS: Anxiety	0.99	ns	1.22	*p* = 0.039
DASS: Stress	1.07	ns	1.59	*p* = 0.028
NASA-TLX: Subscale Average	1.10	*p* = 0.039	1.05	ns
Cortisol: waking	1.15	ns	1.01	ns
Cortisol: +30 min	1.41	*p* = 0.047	1.31	ns
Cortisol: bedtime	2.76	ns	-	-
Testosterone: waking	0.98	ns	0.99	ns
Testosterone: +30 min	0.99	ns	0.99	ns
Testosterone: bedtime	0.99	ns	0.99	ns
Step count	1.00	ns	1.00	ns

## Data Availability

All data pertaining to this study is included in the manuscript.

## Supplementary Materials

The following are available online at https://www.mdpi.com/article/10.3390/ijerph19127271/s1, Table S1: Recruit characteristics at week 1 of BMT, Table S2: Estimated cost of training wastage during basic military training.
