# Supplementary material for: Factors Predicting Training Delays and Attrition of Recruits during Basic Military Training
Source: Int J Environ Res Public Health. 2022 Jun 14;19(12):7271. doi: 10.3390/ijerph19127271 (PMC9223722; doi:10.3390/ijerph19127271)
Supplement: Supplementary file 1 [file ijerph-19-07271-s001.zip › ijerph-1746877-supplementary.pdf]

Supplementary Table S1: Recruit characteristics at week 1 of BMT.

Characteristics	ON-P	DELCOM	DIS
N	36	7	3
Men, *n* (%)	31 (86%)	5 (71%)	1 (33%)
Age, year	24.1 ± 6.8	27.7 ± 9.1	23.3 ± 6.8
Height, cm	178.0 ± 9.7	170.6 ± 11.5	166.7 ± 4.2
Weight, kg	76.0 ± 14.6	73.7 ± 18.1	70.2 ± 11.2
BMI (kg/m^2)	23.9 ± 3.3	25.0 ± 3.4	25.2 ± 3.1
Injuries, *n*	11	5	1
Recruits with injuries, *n*	6 (16.7%)	5	1
**Objective measures**			
Predicted $\dot{V}O_{2max}$, $mL \cdot kg^{-1} \cdot min^{-1}$	43.2 ± 4.4	42.1 ± 3.3	37.4 ± 2.1 *
Push ups completed, *n*	32.8 ± 10.9	30.8 ± 8.4	10.0 ± 8.2 *†
<u>Cortisol (ng/mL)</u>			
Waking	4.9 ± 1.9	4.2	6.9
30 min post-waking	6.4 ± 2.4	6.9	8.6
Bedtime	0.6 ± 0.4	0.7 ± 0.4	0.3
<u>Testosterone (pg/mL)</u>			
Waking	180.4 ± 51.5	156.0 ± 86.2	161.3
30 min post-waking	158.6 ± 45.9	171.7 ± 83.2	143.9
Bedtime	109.6 ± 41.8	106.8 ± 38.6	87.8
Steps per week, *n*	108,608 ± 16,074	82,376 ± 28,121	79984
**Subjective measures**			
<u>Short Stress Recovery Scale</u>			
Stress Composite	7.7 ± 4.0	7.8 ± 4.1	15.3 ± 0.6 *†
Recovery Composite	13.8 ± 3.8	11.0 ± 3.7	11.0 ± 1.0
NASA-TLX Average	55.4 ± 9.3	51.0 ± 10.3	52.0 ± 20.0
<u>Fatigue</u>			
Pre-sleep fatigue	4.0 ± 1.0	3.5 ± 0.7	4.6 ± 1.3
Post-sleep fatigue	3.9 ± 1.0	3.7 ± 0.9	3.9 ± 0.9
<u>DASS-21</u>			
Depression	7.2, 4.0 ± 10	4.4, 4.0 ± 4	18, 24.0 ± 26 †
Anxiety	8.9, 8.0 ± 12	5.2, 4.0 ± 6	14.7, 4.0 ± 6
Stress	12.2, 10.0 ± 16	12.4, 14.0 ± 4	22.0, 22.0 ± 4

Values are mean ± SD except for DASS-21: mean, median ± interquartile range. DASS: Depression Anxiety and Stress Scale; DELCOM: delayed completion of BMT; DIS: discharged from BMT; ON-P: on-pathway, completed BMT on time, NASA-TLX: NASA Task Load Index. * $p < 0.05$ vs on-pathway, † $p < 0.05$ vs delayed completion.

Supplementary Table S2: Estimated cost of training wastage during basic military training.

Training Outcome	%	Incidence per 1000	Cost per Recruit	Cost
Discharge ^	10	100	$47,000	$4,700,000.00
Delayed completion &	9	90	$5,100	$ 459,000.00
			**Total**	**$5,159,000.00**

^ Cost of discharge: estimated recruitment and training cost = $47,000 (AUD) per recruit based on cost estimates by Rudzki et al (1999) and adjusted for inflation to 2021. The full cost of the 12-week BMT course has been attributed to discharge as there is no ability to replace the discharged recruit with a new recruit into that BMT session. & Delayed completion was assumed to be two additional weeks of training per recruit.
